# An improved empirical bayes approach to estimating differential gene expression in microarray time-course data: BETR (Bayesian Estimation of Temporal Regulation)

**DOI:** 10.1186/1471-2105-10-409

**Published:** 2009-12-10

**Authors:** Martin J Aryee, José A Gutiérrez-Pabello, Igor Kramnik, Tapabrata Maiti, John Quackenbush

**Affiliations:** 1 Department of Biostatistics, Harvard School of Public Health, 655 Huntington Avenue, Boston, Massachusetts 02115, USA; 2 Department of Immunology and Infectious Diseases, Harvard School of Public Health, 655 Huntington Avenue, Boston, Massachusetts 02115, USA; 3 Department of Statistics and Probability, Michigan State University, East Lansing, Massachusetts 48824, USA; 4 Department of Biostatistics and Computational Biology and Department of Cancer Biology, Dana-Farber Cancer Institute, 44 Binney St, Boston, Massachusetts 02115, USA

## Abstract

**Background:**

Microarray gene expression time-course experiments provide the opportunity to observe the evolution of transcriptional programs that cells use to respond to internal and external stimuli. Most commonly used methods for identifying differentially expressed genes treat each time point as independent and ignore important correlations, including those within samples and between sampling times. Therefore they do not make full use of the information intrinsic to the data, leading to a loss of power.

**Results:**

We present a flexible random-effects model that takes such correlations into account, improving our ability to detect genes that have sustained differential expression over more than one time point. By modeling the joint distribution of the samples that have been profiled across all time points, we gain sensitivity compared to a marginal analysis that examines each time point in isolation. We assign each gene a probability of differential expression using an empirical Bayes approach that reduces the effective number of parameters to be estimated.

**Conclusions:**

Based on results from theory, simulated data, and application to the genomic data presented here, we show that BETR has increased power to detect subtle differential expression in time-series data. The open-source R package *betr *is available through Bioconductor. BETR has also been incorporated in the freely-available, open-source MeV software tool available from http://www.tm4.org/mev.html.

## Background

The analysis of microarray time-course data presents a number of challenges. First, microarray gene expression data has an inherent complexity due to its high dimensionality and hidden correlations driven by co-expression of genes in biological networks and other factors. Added to this is the fact that additional correlations exist between time points, but time-course sampling is often sparse and irregular due to experimental constraints. Further, temporal processes governing gene expression in cells operate on a wide range of different time scales, making any sampling less than optimal for some applications.

When analyzing microarray gene expression data in general, and time-course experiments in particular, two common goals are to identify genes with similar expression profiles (often using clustering approaches) and to identify those that are differentially expressed across conditions such as disease states. Most commonly used techniques are extensions of methods developed for static (non time-course) experiments. They ignore the sequential nature of time-course data and the resulting time-dependent correlation structure. Analysis methods tailored to time-course data make use of this additional information, improving power to draw conclusions from the data.

Several linear modeling approaches designed for non-time-course experiments [1-5] can be extended for use in time-course experiments. These linear modeling frameworks allow traditional ANOVA analysis but are not well suited to time-course data as they treat time points as unordered. Ignoring the information contained in the sequential sampling leads to a loss of power to detect differentially expressed genes.

Efron et al. [6] and Eckel et al. [7] develop an empirical Bayes framework for detecting differentially expressed genes. Although these methods allow for timecourse data they do not address the non-uniform serial correlation between time points. Guo et al. [8] offer an estimating equation technique that handles serial correlation, but as formulated it can only be used to identify differentially expressed genes in a single-condition time-course. Tai et al. [9] construct a multivariate empirical Bayes statistic applicable to single- and multiple-condition data with variance stabilization for gene-specific covariance matrices.

An alternative approach involves fitting curves to the data [10-12] and performing statistical tests on the smoothed curves. This has the advantage of allowing for different sampling time points between the conditions being compared. A disadvantage is the difficulty in fitting curves to data from experiments with few sampling times.

Here we present BETR (Bayesian Estimation of Temporal Regulation), a novel technique to identify differentially expressed genes that overcomes many of the limitations of existing methods. Our approach explicitly uses the time-dependent structure of the data, employing an empirical Bayes procedure to stabilize estimates derived from the small sample sizes typical in microarray experiments. It is applicable to one- or two-color replicated microarray data, and can be used to detect differences between two conditions or changes from baseline in a single condition.

## Methods

In building a model for time-course data, we decompose the variability in our experimental measurements into its component parts, most importantly time effects, treatment (experimental group) effects and random technical and biological noise. The aim of a two-group experiment is usually to identify genes with a treatment effect as manifested in a significant difference between the groups' individual expression profiles.

**Figure 1:**
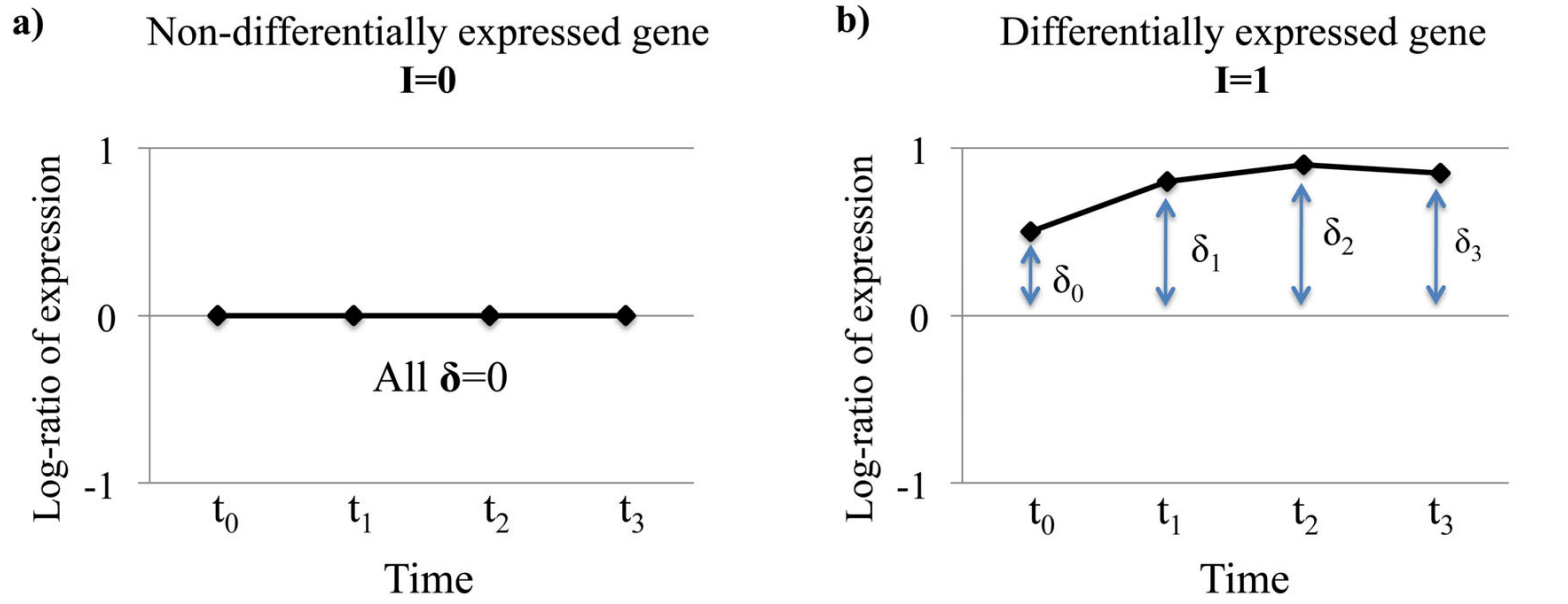
**Time-course expression profiles of two illustrative genes**. Log-ratio of expression between two treatment groups for a) a gene without differential expression, and b) an illustrative differentially expressed gene. I is an indicator of differential expression. **δ **represents the log-fold change at the four time points.

Figure 1 shows the log-ratio of the expression profiles for two illustrative genes in a two-group time-course experiment. Figure 1a represents an idealized gene without differential expression, where the log-ratio of expression between groups is zero at all time points. Any deviation from the flat line in such a gene is due to random noise. In contrast, the gene in figure 1b is differentially expressed, with the treatment effects, **δ **representing the log-ratio between the treatment groups at each time point.

Time-course data is a special case of repeated measures data with the distinguishing feature that the data points are ordered. A key result of this ordering is that correlation between time points in non-uniform. For example, it is often the case that measurements at consecutive time points, such as t_1 _and t_2_, are more highly correlated than those at non-consecutive time points, such as t_1 _and t_4_. BETR takes advantage of the time series structure of the data by allowing correlation between the magnitude of differential expression at different time points; values of **δ **that are close in time are likely to be more correlated than those with greater separation. The data from all genes is used jointly to estimate parameters representing the covariances between time points, as well as gene-specific error terms.

After estimating the model variance parameters, we fit two models to each gene. The simpler model assumes identical mean profiles between conditions (Figure 1a), while the second allows for differential expression (Figure 1b). Bayes' rule is then used to calculate the probability that the gene's data comes from each of these models.

As an intuitive example of the benefit of taking the ordering of time points into account, consider a gene where the differential expression and random noise are of similar magnitude. When analyzing each time point independently the signal will often be masked by the background noise. In contrast, the true signal should have a detectable correlation across time points, making its identification possible.

### Model formulation

Let *I*_*g *_be a Bernoulli (0 or 1) random variable indicating whether gene *g *is differentially expressed across conditions. Our interest is in estimating the probability of differential expression for each gene, given the data (the posterior probability, in Bayesian terminology). We will first describe BETR as applied to a two-group comparison using single channel (Affymetrix) gene expression data, denoting the two experimental conditions as treatment (Tx) and control (Co). At least two replicates are required in each experimental group, although the sample sizes (*N*^*Tx *^and *N*^*Co*^) need not be balanced. Let **X**_*gi *_= (*X*_*gi*1_,..., *X*_*giT*_) denote the log transformed expression values for replicate *i *of gene *g *at the *T *time points:

where  and  represent gene *g*'s mean expression in the two groups. The error terms **e**_*gi *_are assumed to be multivariate normal, *MVN*_*T *_(**0**, Σ_*Eg*_), random effects with a compound symmetry covariance structure. This relatively simple two-parameter covariance structure allows for within-replicate correlation between the errors at different time points accounting for biological or experimental replicate effects in the case of repeated measurements on the same experimental unit.

Our aim is to determine if there is a difference in expression between the groups, that is, whether . Although we allow different numbers of replicates in the two groups we will here, for the sake of simplicity, assume a common number *N *= *N*^*Tx *^= *N*^*Co*^. Within each group we define  and , the mean observed expression profile and mean error across replicates respectively. We model , the log ratio of expression in the two conditions.

We define **δ**_*g *_= (*δ*_*g*1_,...,*δ*_*gT*_) to be the vector of log-ratios at each time point. A notable feature of BETR is that the parameters of interest **δ**_*g *_are modeled as random effects rather than fixed effects for differentially expressed genes. In statistical models fixed effects are typically used for variables of interest, such as differences between experimental groups, or between time points. Random effects are usually used to account only for the remaining sources of variation that are not of interest, such as random technical measurement error or biological variability between subjects in the same treatment group. By modeling the **δ**_*g *_as random effects, we are able to capture non-uniform correlation between them that arises from the time-course structure of the data.

We define the indicator *I*_*g *_to describe whether or not a gene is differentially expressed. For those genes without differential expression **δ**_*g *_is modeled as a mean zero fixed effect at all time points. In the case of a differentially expressed gene (*I*_*g *_= 1), we model **δ**_*g *_as a random effect, allowing for non-zero log-ratios.

By modeling the log-ratio as a non-zero realization of a random effect we allow correlation between the magnitude of differential expression at different time points.

It follows that the distribution of the gene's data points, **Y**_*g*_, takes on different forms depending on whether the gene is differentially expressed or not:

By considering which of the two distributions above better fit the gene's data, we can make an inference about the probability of differential expression using Bayes' rule:

where *p *represents the proportion of differentially expressed genes (see next section for estimation). We report as differentially expressed those genes whose probability of differential expression, *P*(*I*_*g *_= 1|**Y**_*g*_) is greater than a user-defined threshold 1 - *α*.

### Parameter estimation

In the above model the proportion of differentially expressed genes, *p*, and the two components of variance, Σ_*Eg *_and Σ_*Dg *_are unknown. Σ_*Eg *_represents the sample variance about the treatment group mean and is estimated using the pooled sample covariance  where  and likewise for *S*^*Co*^. When sample sizes are small we recommend constraining the structure of Σ_*Eg *_to be compound symmetric, requiring the estimation of only two parameters; the variance and covariance terms are obtained by averaging the diagonal and off-diagonal terms respectively. To further lessen the impact of small sample sizes the variance and covariance estimates are stabilized using the empirical Bayes shrinkage procedure of Smyth [5].

The second covariance parameter, Σ_*Dg*_, relates to the primary quantity of interest, the magnitude of differential expression. Since we model the differential expression vector as correlated random effects with known mean 0, we can estimate Σ_*Dg *_by the sample covariance matrix which simplifies to *S*_*Dg *_= **Y**_*g *_. The gene-specific estimates are stabilized using a modified version of the empirical Bayes matrix shrinkage procedure introduced by Tai and Speed [9,13]. The Σ_*Dg *_are estimated by , shrinkage estimators calculated as a weighted average of the gene specific *S*_*Dg *_and a target covariance matrix. Since Σ_*Dg *_is non-zero only for differentially expressed genes, we base our target matrix only on the mean of *S*_*Dg *_for those genes where the probability of differential expression,  = *P*(*I*_*g *_= 1 **Y**|_*g*_), is greater than the user-defined significance cutoff, 1 - *α*. The fraction of genes where  ≥ 1 - *α *is used to estimate *p*, the proportion of differentially expressed genes.

In the parameter estimation procedure described above, estimation of the covariance parameters, Σ_*Dg*_, depends on knowledge of the *I*_*g*_, which in turn depend on knowledge of the covariance parameters and the proportion, *p*, of differentially expressed genes. We therefore use an iterative procedure that alternates between updating the *I*_*g *_estimates and the Σ_*Dg *_estimates until the process converges. The process starts with an initial default estimate of *p *and a rough gene ranking obtained for example by ANOVA. Σ_*Dg *_is estimated using an initial target covariance matrix derived from the mean *S*_*Dg *_of the top ranked genes. Given the Σ_*Dg *_estimates we then obtain a rough first iteration estimate of  using equation 1. Those genes with  >1 - *α *are used to construct a new shrinkage target matrix allowing a seconditeration estimation of Σ_*Dg*_. We have found that the  estimates typically stabilize within 3-6 iterations. The final estimates 1 -  can then be thought of as roughly analogous to a p-value. These pseudo p-values are adjusted for multiple comparisons using Storey's positive false-discovery rate (pFDR) procedure [14].

### Two-color microarray data

When using two color microarrays two samples are co-hybridized to each chip, and data is obtained in the form of expression ratios between conditions. Let *X*_*gi *_represent the log ratio of expression of gene *g *at T time points for replicate *i*. We then express *Y*_*g *_the average log ratio, similarly to the one color model above:

The rest of the procedure is identical to that for the one-color case.

### Method Evaluation

To assess the performance of BETR we compared it to three established methods, the linear model approach with variance shrinkage implemented in the R/Bioconductor package limma [5], the spline-based method implemented in EDGE (Extraction and analysis of Differential Gene Expression, version 1.1.290) [10,15], and the twosample multivariate empirical Bayes (MB) statistic[9]implemented in the R/Bioconductor package timecourse. We compared performance of the methods using data from an experiment investigating host response to tuberculosis (TB) infection in mouse and a simulated dataset derived from the mouse TB data.

#### Mouse TB host-response data

Tuberculosis is a significant and growing public health problem, with an estimated two billion people infected worldwide and increasing concern about multi-drug resistant strains. Despite its prevalence, only about ten percent of people infected with *Mycobacterium tuberculosis *(MTB) progress to active disease. Host genetic factors that influence the outcome of TB infection have been identified both in humans and mouse models of the disease. The known factors only explain a fraction of the variability observed in the host reaction to infection. To better understand host genetic factors and their impact on the dynamics of infection response, we analyzed gene expression in a mouse model of TB infection using C57BL/6 and C3H.B6-sst1 mice, resistant and susceptible, respectively, to infection. Bone marrow-derived macrophages were extracted from three mice of each strain, primed with interferon gamma and then infected with MTB *in vitro*. RNA was extracted for gene expression analysis on Affymetrix 4302 microarrays at four time points: prior to infection (0 hours) and 6, 30 and 78 hours post infection. Details are given in Additional File 1: Supplementary Methods. The raw CEL files were normalized using the Li-Wong method [16,17]. A filter was applied to remove probe sets with constant expression across all arrays, defined as an intensity range of less than 500. The resulting 10,042 probe data set representing 6,458 genes was analyzed using the four time-course tools. Experimental data is available from ArrayExpress (accession # E-MTAB-90).

#### Simulated Data

Although simulated data may fail to capture all of the features of and correlations within gene expression data, it is useful for understanding the properties of a new analytical method. Simulated data has the advantage that we know the 'truth' and allows us to compare the performance of different methods helping us broadly define the conditions under which particular methods are most suitable. A major difficulty in simulating microarray data sets lies in our lack of understanding of the true correlation structure of such data. This includes the correlation between genes and in the case of time-course data, the correlation between successive time points. To address these concerns, we began with the TB data and randomly selected 2000 genes that were expressed above background in the C57BL/6 resistant strain, thus preserving some of the correlations in the real data. To create data for the second condition, we then selected 100 genes, shifted their expression levels by 1.5- to 3-fold at 1 to 4 time points, and added random Gaussian noise with a mean of 1.5 fold to the expression levels for all 2000 genes.

## Results and Discussion

In order to identify the set of differentially expressed genes in an experiment genes are ranked in order of decreasing evidence for differential expression and a cutoff is chosen that balances the numbers of false positives and false negatives. A receiver operating characteristic (ROC) curve plots the true positive rate as a function of the false positive rate as the cutoff is changed and can be used to assess the performance of the ranking criteria. For simulated data where it is known which genes are differentially expressed, ROC analysis is possible. Consequently, we analyzed each simulated dataset using limma, EDGE, the MB-statistic and BETR, and evaluated their ROC performance. In limma we fit an eight coefficient linear model to model the two conditions and four time points. Genes were ranked according to the moderated F-test of the four between-strain contrasts [5]. The corresponding EDGE analysis was carried out using the default natural cubic spline basis and choosing the option to include the baseline levels in analysis. The MB-statistic was calculated using the R package timecourse with default options.

### Analysis of simulated data

**Figure 2:**
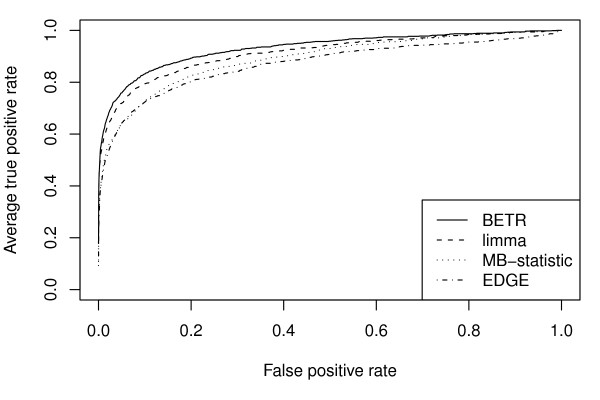
**Performance assessment: ROC curves**. ROC curves showing the true positive/false positive rates for detecting differentially expressed genes using simulated data.

Figure 2 shows the results of analyzing a four time point simulated dataset comparing two experimental conditions. Five percent of the genes are differentially expressed, with three or four time points affected by a 1.5- to 3-fold change. The graph shows the average of ten simulations. The power, or true positive rate, (y-axis) can be thought of as the probability that a truly differentially expressed gene will be correctly identified as such. BETR has greater power to detect differentially expressed genes with the most pronounced benefit at low false positive rates in the commonly used range of 5-20%. At a false positive rate of 5%, for example, BETR successfully identifies 65% of the differentially expressed genes, compared to 62% for limma, 56% for the MBstatistic and EDGE.

To test the hypothesis that BETR's advantage would be most pronounced in the case of noisy data with small but sustained effects, we characterized BETR's performance under a variety of different conditions, varying the duration of differential expression. In each case we chose a cutoff to achieve a false positive rate of 5% and assess the power to correctly identify differentially expressed genes (the true positive rate). The results presented are the average of ten simulations.

**Figure 3:**
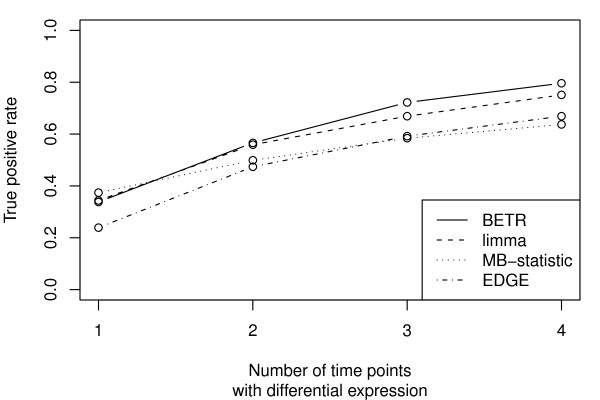
**Performance assessment: True positive rate vs. number of time points with differential expression**. True positive rate as a function of the number of the four time points with differential expression. The significance cutoff is chosen to maintain a false positive rate of 5%.

We estimate the power to detect differentially expressed genes as a function of the number of time points with differential expression, ranging from a short spike at a single time point to differential expression across all four time points (Figure 3). The true positive rate for each method was read from its ROC curve. When only a single time point has differential expression we gain nothing by considering the order of the time points and as a result, there is no benefit to using BETR. The relative power of BETR improves as a function of the number of time points at which a gene is differentially expressed and it dominates the other methods when there is differential expression at 3 or 4 time points.

### Analysis of mouse TB host-response data

The tuberculosis infection time-course experiment was analyzed with each of the four methods to detect differentially expressed genes. For BETR, limma and EDGE we chose a cutoff to obtain an estimated false discovery rate of 1%. Since there is currently no way to estimate false discovery rates with the MB-statistic the size of this gene list was chosen for the purposes of comparison to be the average of the other methods. The union of the four lists contained 528 genes of which only 146 were common to all methods.

To investigate the differences between the results produced by the four methods we used Gene Set Enrichment Analysis (GSEA) to identify Gene Ontology terms and Kyoto Encyclopedia of Genes and Genomes (KEGG) pathways that show concordant differences between the strains [18,19]. In each case the full ranked gene list was used as input to GSEA. Gene sets with fewer than 15 or more than 500 genes were excluded. Genes with multiple probe sets were represented by the most significant.

Each of the four methods is equally capable of detecting pronounced differences. For example, the core set of enriched categories that are consistently ranked highly by all the methods includes the KEGG pathway *cell adhesion molecules *(p < 0.05; Additional File 1: Supplemental Tables S1-S4). These proteins play a key role during the immune response through their mediation of physical cell interactions. A key mechanism of tuberculosis control is the formation of inflammatory lesions called granulomas. These cellular aggregates, consisting largely of macrophages and lymphocytes, serve to contain and destroy the tuberculosis bacteria [20]. Reduced adhesion molecule activity has been shown to lead to significant reduction in lymphocyte recruitment to the lungs and inability to form well-defined granulomas, leading to dissemination of bacteria throughout the lungs and hastened death [21,22].

A related and particularly notable gene set that is uniquely identified by BETR is the KEGG pathway *leukocyte transendothelial migration*. Factors that govern immune cell migration can significantly influence the effectiveness of host response to tuberculosis infection through their role in recruitment of cells to sites of granuloma formation [23]. One of the key distinguishing features of tuberculosis disease in susceptible mice is their reduced capacity to form effective granulomas, possibly related to their inability to localize leukocytes within the lung [24]. A successful granulomatous response depends on correct cellular composition and local organization, both of which are sensitive to disruptions in leukocyte migration. Structural deficiencies in granuloma architecture have been identified as a factor underlying heightened susceptibility to tuberculosis [25]. The core set of genes driving significance of the leukocyte endothelial migration gene set includes the matrix metallopeptidase MMP-9 which has been shown to be an essential component of resistance through its roles in the cell recruitment and tissue remodeling required for the formation of well organized granulomas [26]. Interestingly, the Wnt signaling pathway, also uniquely identified by BETR, has recently been shown to play an important role in recruiting T cells to sites of inflammation through direct induction of MMP-2 and MMP-9 [27].

**Figure 4:**
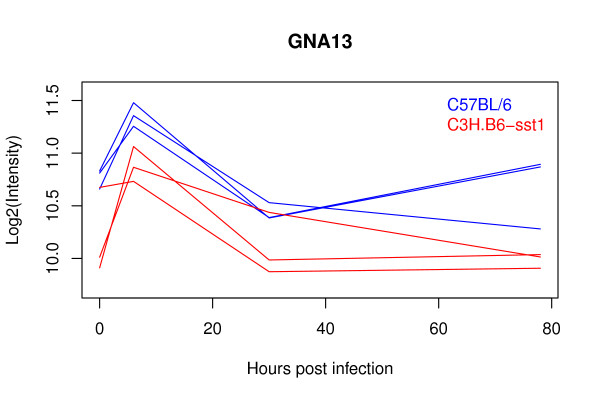
**An example of a gene uniquely identified by BETR**. BETR has greater power than existing methods to detect genes with subtle and noisy differential expression patterns that are sustained over time. GNA13 is expressed at subtly higher levels in the TB resistant C57BL/6 mice compared to the more susceptible C3H.B6-sst1 mice.

The distinguishing feature of the genes uniquely identified by BETR is a small and/or noisy differential expression signal that is sustained over several time points. An example is guanine nucleotide binding protein, alpha 13 (GNA13) shown in Figure 4. The GNA13 protein has been found to play a key role in leukocyte migration [28] and is expressed at subtly higher levels in the C57BL/6 (resistant) mice compared to the C3H.B6-sst1 (susceptible) mice with an average difference of 1.4 fold. The improved power of BETR to detect such genes reveals leukocyte migration as an interesting process that plays a potential role in mediating the strain-specific differences in ability to control tuberculosis.

## Conclusions

Based on simulated data our proposed method, BETR, outperforms three commonly used techniques in the analysis of time-course data. This advantage is particularly noticeable for genes with a small but sustained differential expression signal. When the magnitude of differential expression is of similar magnitude to background noise, it is difficult to identify by examining each time point in isolation. These patterns of differential expression become easier to identify when the time series structure of the data is taken into account; a small, noisy signal becomes identifiable if it is sustained across several adjoining time points.

While BETR has no advantage when the differential expression signal is transient, its relative performance improves as the signal is sustained over additional time points. This improvement is due to the fact that BETR accounts for the correlation between successive time points. The significance of this correlation increases as a function of the number of differentially expressed time points increases. Analysis of the mouse TB host-response data confirms that our method has greater power to detect such sustained differences in a real dataset. We identified a set of genes involved in cell homing during immune response that was not detected by the other methods. Several genes in this class respond with small expression changes whose significance is only apparent when their sustained nature is taken into account. These results suggest that poor control of tuberculosis infection is in part driven by deficient regulation of cell migration factors, resulting in poor granuloma formation and subsequent inability to limit bacterial growth.

An inherent challenge in genomic data analysis is identifying effects that are robust yet subtle. Based on results from theory, simulated data, and application to the genomic data presented here, we expect BETR to outperform existing methods under these circumstances. This increased sensitivity has the potential to elucidate important biological themes that may otherwise go unobserved.

## Authors' contributions

TM and MA conceived of the statistical technique. MA developed and implemented the technique and drafted the manuscript. IK conceived of the mouse model microarray experiments and helped interpret the findings. JG performed the microarray experiments. JQ participated in the conception and coordination of the project and helped draft the manuscript. All authors read and approved the final manuscript.

## Supplementary Material

Additional file 1 **Supplementary methods and tables**. This file includes additional information on the methods used and supplemental tables supporting the results presented in the paper. Click here for file
