# Supplementary material for: An improved empirical bayes approach to estimating differential gene expression in microarray time-course data: BETR (Bayesian Estimation of Temporal Regulation)
Source: BMC Bioinformatics. 2009 Dec 10;10:409. doi: 10.1186/1471-2105-10-409 (PMC2801687; doi:10.1186/1471-2105-10-409)
Supplement: Additional file 1 — Supplementary methods and tables. This file includes additional information on the methods used and supplemental tables supporting the results presented in the paper. [file 1471-2105-10-409-S1.DOC]

**Supplementary Methods**

Isolation and MTB infection of murine bone marrow-derived macrophages (BMDM) in vitro

BMDM were isolated from femurs and tibias of mice (6–8 weeks old). The cells were cultured in a complete culture medium mixed with 50% DMEM and 50% HAM F-12 containing 10% FCS (HyClone). Macrophage precursors were enriched by culturing in the medium supplemented with 1 ng/ml IL-3 (Sigma-Aldrich) and 20% of L-929 fibroblast-conditioned medium followed by removal of more differentiated plastic adherent cells. After a 2-day culture, the non-adherent cells were collected and cultured in medium containing 40% L929-conditioned medium for 4 days to induce their differentiation into macrophages. Subsequently the cells were culture in 75 cm2 flasks in complete medium containing 20% of L-929-conditioned medium to form monolayers.  Recombinant interferon-gamma (50 U/ml) was added to the medium and after 24h the BMDM monolayers were infected with MTB at a multiplicity of infection (MOI) 1 bacteria per macrophage (MOI 1:1). The cells were washed by PBS with 1% FCS at 6 h post infection and incubated in complete medium for indicated times.

RNA isolation and microarray protocol

To collect RNA macrophage monolayers were washed with PBS three times and lysed in Trizol (Invitrogen). RNA isolation and hybridization to Affymetrix Mouse 4302 GeneChip microarrays was carried out according to according to the manufacturer’s protocol.

| **KEGG** | **# genes** | **p-value** |
| --- | --- | --- |
| HSA03010_RIBOSOME | 50 | 0.00000 |
| HSA04310_WNT_SIGNALING_PATHWAY | 59 | 0.00400 |
| HSA04520_ADHERENS_JUNCTION | 40 | 0.00600 |
| HSA03050_PROTEASOME | 22 | 0.01101 |
| HSA00620_PYRUVATE_METABOLISM | 19 | 0.01911 |
| HSA05010_ALZHEIMERS_DISEASE | 18 | 0.02102 |
| HSA00280_VALINE_LEUCINE_AND_ISOLEUCINE_DEGRADATION | 25 | 0.02503 |
| HSA04514_CELL_ADHESION_MOLECULES | 30 | 0.04200 |
| HSA04670_LEUKOCYTE_TRANSENDOTHELIAL_MIGRATION | 47 | 0.04400 |
|  |  |  |
| **GO** | **# genes** | **p-value** |
| STRUCTURAL_CONSTITUENT_OF_RIBOSOME | 62 | 0.00000 |
| MITOCHONDRIAL_INNER_MEMBRANE | 50 | 0.00000 |
| MITOCHONDRIAL_ENVELOPE | 70 | 0.00000 |
| MITOCHONDRIAL_RESPIRATORY_CHAIN | 21 | 0.00100 |
| CELLULAR_COMPONENT_DISASSEMBLY | 17 | 0.00100 |
| CYTOSKELETON_ORGANIZATION_AND_BIOGENESIS | 92 | 0.00300 |
| MITOCHONDRIAL_PART | 100 | 0.00300 |
| MITOCHONDRIAL_MEMBRANE_PART | 40 | 0.00400 |
| RECEPTOR_SIGNALING_PROTEIN_SERINE_THREONINE_KINASE_ACTIVITY | 15 | 0.00402 |
| MITOCHONDRIAL_MEMBRANE | 64 | 0.00500 |
| OXIDOREDUCTASE_ACTIVITY__ACTING_ON_NADH_OR_NADPH | 16 | 0.00503 |
| CYTOSKELETON | 144 | 0.00600 |
| HETEROCYCLE_METABOLIC_PROCESS | 15 | 0.00603 |
| RIBOSOME | 30 | 0.01000 |
| RIBOSOMAL_SUBUNIT | 16 | 0.01605 |
| REGULATION_OF_HYDROLASE_ACTIVITY | 38 | 0.01700 |
| POSITIVE_REGULATION_OF_MAP_KINASE_ACTIVITY | 16 | 0.01804 |
| GLYCOPROTEIN_BIOSYNTHETIC_PROCESS | 28 | 0.02100 |
| ELECTRON_CARRIER_ACTIVITY | 39 | 0.02300 |
| OXIDOREDUCTASE_ACTIVITY | 121 | 0.02300 |
| AMINO_ACID_AND_DERIVATIVE_METABOLIC_PROCESS | 37 | 0.02400 |
| CYTOSKELETAL_PART | 93 | 0.02500 |
| MONOVALENT_INORGANIC_CATION_TRANSMEMBRANE_TRANSPORTER_ACTIVITY | 15 | 0.02920 |
| ORGANELLAR_RIBOSOME | 18 | 0.03006 |
| POSITIVE_REGULATION_OF_HYDROLASE_ACTIVITY | 23 | 0.03307 |
| PROTEIN_SERINE_THREONINE_PHOSPHATASE_ACTIVITY | 15 | 0.03310 |
| MITOCHONDRIAL_RIBOSOME | 18 | 0.03407 |
| GLYCOPROTEIN_METABOLIC_PROCESS | 40 | 0.03600 |
| UDP_GLYCOSYLTRANSFERASE_ACTIVITY | 16 | 0.03618 |
| MICROTUBULE_CYTOSKELETON | 66 | 0.03800 |
| PHOSPHOLIPID_METABOLIC_PROCESS | 24 | 0.04000 |
| ELECTRON_TRANSPORT | 15 | 0.04221 |
| POSITIVE_REGULATION_OF_CATALYTIC_ACTIVITY | 63 | 0.04500 |

**Table S1**: KEGG pathway and Gene Ontology gene sets identified as differentially expressed by GSEA on the ranked list of genes generated by the BETR algorithm

| **KEGG pathway** | **# genes** | **p-value** |
| --- | --- | --- |
| HSA03010_RIBOSOME | 50 | 0.00000 |
| HSA04120_UBIQUITIN_MEDIATED_PROTEOLYSIS | 26 | 0.00502 |
| HSA04514_CELL_ADHESION_MOLECULES | 30 | 0.03103 |
| HSA00860_PORPHYRIN_AND_CHLOROPHYLL_METABOLISM | 16 | 0.03262 |
| HSA00190_OXIDATIVE_PHOSPHORYLATION | 90 | 0.03900 |
| HSA04150_MTOR_SIGNALING_PATHWAY | 23 | 0.04623 |
|  |  |  |
| **GO** | **# genes** | **p-value** |
| STRUCTURAL_CONSTITUENT_OF_RIBOSOME | 62 | 0.00000 |
| STRUCTURAL_MOLECULE_ACTIVITY | 93 | 0.00000 |
| CYTOSKELETAL_PART | 93 | 0.00000 |
| CYTOSKELETON | 144 | 0.00000 |
| ACTIN_CYTOSKELETON | 57 | 0.00100 |
| TRANSLATION | 89 | 0.00100 |
| INTRACELLULAR_NON_MEMBRANE_BOUND_ORGANELLE | 278 | 0.00100 |
| TRANSMEMBRANE_RECEPTOR_ACTIVITY | 81 | 0.00300 |
| CELL_CYCLE_PROCESS | 104 | 0.00300 |
| NON_MEMBRANE_BOUND_ORGANELLE | 278 | 0.00300 |
| EXTRACELLULAR_REGION | 97 | 0.00400 |
| M_PHASE_OF_MITOTIC_CELL_CYCLE | 51 | 0.00500 |
| MITOSIS | 50 | 0.00601 |
| MICROTUBULE_CYTOSKELETON | 66 | 0.00800 |
| EXTRACELLULAR_SPACE | 58 | 0.00900 |
| MITOTIC_CELL_CYCLE | 90 | 0.00900 |
| TISSUE_DEVELOPMENT | 24 | 0.00901 |
| M_PHASE | 56 | 0.01100 |
| ECTODERM_DEVELOPMENT | 15 | 0.01230 |
| EXTRACELLULAR_REGION_PART | 68 | 0.01300 |
| CELL_CYCLE_GO | 169 | 0.01400 |
| CELL_CYCLE_PHASE | 90 | 0.01600 |
| CELLULAR_BIOSYNTHETIC_PROCESS | 151 | 0.01700 |
| MACROMOLECULE_BIOSYNTHETIC_PROCESS | 138 | 0.01900 |
| SPINDLE | 27 | 0.01904 |
| REGULATION_OF_MITOSIS | 24 | 0.01906 |
| POSITIVE_REGULATION_OF_TRANSCRIPTION_FROM_RNA_POLYMERASE_II_PROMOTER | 23 | 0.02209 |
| CYTOKINE_BINDING | 24 | 0.02307 |
| BIOSYNTHETIC_PROCESS | 203 | 0.02600 |
| SUBSTRATE_SPECIFIC_TRANSPORTER_ACTIVITY | 108 | 0.02800 |
| MEMBRANE_FUSION | 17 | 0.02932 |
| CYTOPLASMIC_VESICLE | 47 | 0.03100 |
| CHROMOSOME_SEGREGATION | 17 | 0.03480 |
| ORGANELLE_ORGANIZATION_AND_BIOGENESIS | 221 | 0.04100 |
| CELLULAR_DEFENSE_RESPONSE | 24 | 0.04116 |
| TRANSMEMBRANE_RECEPTOR_PROTEIN_KINASE_ACTIVITY | 16 | 0.04162 |
| MICROTUBULE_ORGANIZING_CENTER | 22 | 0.04422 |
| NERVOUS_SYSTEM_DEVELOPMENT | 72 | 0.04500 |
| CYTOPLASMIC_MEMBRANE_BOUND_VESICLE | 46 | 0.04600 |
| TRANSCRIPTION_ACTIVATOR_ACTIVITY | 67 | 0.04600 |
| ESTABLISHMENT_OF_LOCALIZATION | 330 | 0.04800 |
| CYTOSKELETON_ORGANIZATION_AND_BIOGENESIS | 92 | 0.04900 |

**Table S2**: KEGG pathway and Gene Ontology gene sets identified as differentially expressed by GSEA on the ranked list of genes generated by the EDGE algorithm

| **KEGG pathway** | **# genes** | **p-value** |
| --- | --- | --- |
| HSA04514_CELL_ADHESION_MOLECULES | 30 | 0.01301 |
| HSA00564_GLYCEROPHOSPHOLIPID_METABOLISM | 29 | 0.01401 |
| HSA04610_COMPLEMENT_AND_COAGULATION_CASCADES | 20 | 0.01913 |
| HSA03320_PPAR_SIGNALING_PATHWAY | 26 | 0.03912 |
|  |  |  |
| **GO category** | **# genes** | **p-value** |
| ELECTRON_TRANSPORT | 15 | 0.00000 |
| AMINO_ACID_METABOLIC_PROCESS | 30 | 0.00000 |
| INFLAMMATORY_RESPONSE | 51 | 0.00000 |
| TRANSMEMBRANE_RECEPTOR_ACTIVITY | 81 | 0.00000 |
| INTRINSIC_TO_PLASMA_MEMBRANE | 247 | 0.00000 |
| INTEGRAL_TO_PLASMA_MEMBRANE | 245 | 0.00100 |
| AMINO_ACID_AND_DERIVATIVE_METABOLIC_PROCESS | 37 | 0.00200 |
| RESPONSE_TO_WOUNDING | 71 | 0.00200 |
| CELL_CYCLE_PHASE | 90 | 0.00300 |
| TRANSMEMBRANE_RECEPTOR_PROTEIN_KINASE_ACTIVITY | 16 | 0.00305 |
| M_PHASE_OF_MITOTIC_CELL_CYCLE | 51 | 0.00400 |
| OXIDOREDUCTASE_ACTIVITY | 121 | 0.00400 |
| MITOTIC_CELL_CYCLE | 90 | 0.00500 |
| CYTOSKELETON | 144 | 0.00500 |
| MITOSIS | 50 | 0.00501 |
| RECEPTOR_ACTIVITY | 132 | 0.00600 |
| CELL_CYCLE_PROCESS | 104 | 0.00600 |
| PLASMA_MEMBRANE_PART | 289 | 0.00600 |
| M_PHASE | 56 | 0.00900 |
| CARBOXYLIC_ACID_METABOLIC_PROCESS | 66 | 0.00900 |
| ACTIN_CYTOSKELETON | 57 | 0.01000 |
| PROTEIN_TYROSINE_KINASE_ACTIVITY | 21 | 0.01003 |
| CYTOSKELETAL_PART | 93 | 0.01100 |
| PLASMA_MEMBRANE | 384 | 0.01300 |
| NITROGEN_COMPOUND_METABOLIC_PROCESS | 58 | 0.01400 |
| PROTEIN_DNA_COMPLEX_ASSEMBLY | 17 | 0.01418 |
| ORGANIC_ACID_METABOLIC_PROCESS | 66 | 0.01500 |
| METALLOPEPTIDASE_ACTIVITY | 16 | 0.01517 |
| RESPONSE_TO_EXTERNAL_STIMULUS | 114 | 0.01600 |
| BLOOD_COAGULATION | 15 | 0.02037 |
| CELL_MIGRATION | 34 | 0.02102 |
| COAGULATION | 15 | 0.02340 |
| REGULATION_OF_CYTOKINE_BIOSYNTHETIC_PROCESS | 16 | 0.02345 |
| MICROTUBULE_CYTOSKELETON | 66 | 0.02800 |
| CYTOKINE_METABOLIC_PROCESS | 17 | 0.02851 |
| CHROMOSOME__PERICENTRIC_REGION | 21 | 0.02923 |
| CYTOKINE_BIOSYNTHETIC_PROCESS | 17 | 0.03067 |
| G_PROTEIN_COUPLED_RECEPTOR_PROTEIN_SIGNALING_PATHWAY | 71 | 0.03100 |
| CYTOSKELETON_ORGANIZATION_AND_BIOGENESIS | 92 | 0.03100 |
| AMINE_METABOLIC_PROCESS | 51 | 0.03200 |
| GENERATION_OF_NEURONS | 15 | 0.03262 |
| REGULATION_OF_BODY_FLUID_LEVELS | 17 | 0.03265 |
| CELL_PROJECTION | 45 | 0.03600 |
| PHOSPHORIC_ESTER_HYDROLASE_ACTIVITY | 69 | 0.03600 |
| REGULATION_OF_MITOSIS | 24 | 0.03607 |
| EXTRACELLULAR_REGION | 97 | 0.04000 |
| TISSUE_DEVELOPMENT | 24 | 0.04004 |
| POSITIVE_REGULATION_OF_TRANSLATION | 15 | 0.04476 |
| MICROTUBULE_ORGANIZING_CENTER | 22 | 0.04523 |
| CHROMOSOMAL_PART | 58 | 0.04600 |
| INTEGRAL_TO_MEMBRANE | 387 | 0.04700 |
| PROTEIN_HOMODIMERIZATION_ACTIVITY | 53 | 0.04800 |
| PHOSPHORIC_MONOESTER_HYDROLASE_ACTIVITY | 57 | 0.04800 |
| CYTOKINE_BINDING | 24 | 0.04814 |
| CELL_CYCLE | 169 | 0.05000 |

**Table S3**: KEGG pathway and Gene Ontology gene sets identified as differentially expressed by GSEA on the ranked list of genes generated by the limma algorithm

| **KEGG pathway** | **# genes** | **p-value** |
| --- | --- | --- |
| HSA04514_CELL_ADHESION_MOLECULES | 30 | 0.03800 |
|  |  |  |
| **GO category** | **# genes** | **p-value** |
| PROTEASOME_COMPLEX | 22 | 0.00000 |
| SPLICEOSOME | 29 | 0.00000 |
| UNFOLDED_PROTEIN_BINDING | 30 | 0.00000 |
| TRANSMEMBRANE_RECEPTOR_PROTEIN_SERINE_THREONINE_KINASE_SIGNALING_PATHWAY | 28 | 0.01101 |
| CAMP_MEDIATED_SIGNALING | 16 | 0.03333 |
| CELLULAR_DEFENSE_RESPONSE | 24 | 0.03715 |
| G_PROTEIN_SIGNALING__COUPLED_TO_CAMP_NUCLEOTIDE_SECOND_MESSENGER | 15 | 0.03753 |
| MICROTUBULE_ASSOCIATED_COMPLEX | 18 | 0.04036 |
| GUANYL_NUCLEOTIDE_BINDING | 29 | 0.04900 |

**Table S4**: KEGG pathway and Gene Ontology gene sets identified as differentially expressed by GSEA on a gene list ranked by the MB-statistic.
